# Guidelines and best practice recommendations on reproductive health services provision amid COVID-19 pandemic: scoping review

**DOI:** 10.1186/s12889-021-10346-2

**Published:** 2021-02-03

**Authors:** Lemi Belay Tolu, Garumma Tolu Feyissa, Wondimu Gudu Jeldu

**Affiliations:** 1 Department of Obstetrics and Gynecology, Saint Paul’s Hospital Millennium Medical College, Addis Ababa, Ethiopia; 2 Department of Health, Behaviour, and Society, Jimma University, Jimma, Ethiopia

**Keywords:** COVID-19, Pandemics, Reproductive health, Antenatal care, Labor/delivery, Postnatal care, Abortion, Contraception, SARS-CoV-2

## Abstract

**Background:**

Policymakers and health professionals prefer to use summarized evidence of practice recommendations. The aim of this scoping review is therefore to identify available guidelines, consensus statements, the standard of practice, and practice recommendations on reproductive health service provision during the COVID-19 pandemics.

**Methods:**

We searched guideline databases and websites of professional associations and international organizations working on sexual and reproductive health. We looked for practice recommendations on sexual reproductive health services (SRH) during COVID-19 pandemics. Additionally, we searched: MEDLINE, EMBASE, and Google Scholar. Data extraction was done by two independent reviewers using a customized tool that was developed to record the key information of the source that’s relevant to the review question. The difference between the two authors on data extraction was resolved by discussion.

**Results:**

A total of 21 records were included in the review. Identified recommendations were classified into thematic areas. The records addressed approaches to antenatal care, labour and delivery, postnatal care, safe abortion, contraception, gender-based violence, and artificial reproduction.

**Conclusions:**

There were consistent consensus statements and recommendations that there should be access to sexual and reproductive health services like antenatal care (ANC), postnatal care (PNC), contraception service, safe abortion care, and clinical management of rape survivors during the COVID-19 pandemics with the concerted effort of service re-organization. The practice recommendations focus on innovative ways of service provision to minimize patient and staff exposure to COVID-19 as well as alleviate the burden on the health care system. These include utilizing telemedicine and community/home-based care or self-care.

**Supplementary Information:**

The online version contains supplementary material available at 10.1186/s12889-021-10346-2.

## Background

The 2019–20 coronavirus pandemic is an ongoing pandemic of coronavirus disease 2019 (COVID-19), caused by severe acute respiratory syndrome coronavirus 2 (SARS-CoV-2) [1]. The outbreak was first identified in Wuhan, Hubei Province, China, in December 2019. The World Health Organization (WHO) declared the outbreak to be a Public Health Emergency of International Concern on 30 January 2020 and recognized it as a pandemic on 11 March 2020 [2, 3].

The WHO considered reproductive health services, including care during pregnancy and child breath as an essential health service to continue during the COVID-19 pandemics [4]. Additionally, WHO Stated, “Women’s choices and rights to sexual and reproductive health care should be respected irrespective of COVID-19 status, including access to contraception and safe abortion to the full extent of the law” [5]. But when staff and services are under extreme stress there is a real risk of increasing avoidable harm. The tremendous burden caused by the COVID-19 outbreak is exceeding the capacity of many national and local health systems which is jeopardizing routine service delivery and undermining other health priorities. Furthermore, there might be reduced healthcare-seeking behavior among patients because of fear and anxiety of contracting COVID-19 [6, 7] As such the evolving COVID-19 pandemic may affect routine services including sexual, reproductive, and maternal health services delivery. Marie Stopes International (MSI) warned near 9.5 million people will miss out on reproductive service if service reduction continues for 3 months because of the lockdown [8]. Experience in past epidemics also has shown that lack of access to essential health services and shut down of services unrelated to the epidemic response resulted in more deaths than the epidemic itself [9].

The aim of this scoping review is therefore to identify available guidelines, consensus statements, the standard of practice, and practice recommendations on reproductive health service provision during the COVID-19 pandemic.

### Hypothesis

- What’s is the reproductive health service practice approach during the current COVID-19 pandemic?
- What are the available recommendations on service re-organization of reproductive health services amid COVID-19 pandemics?

## Methods and materials

### Types of source and search strategy

We followed three steps in locating relevant records. First, we searched for professional associations and international organization’s guidelines, protocols, consensus statements, and practice recommendations on sexual reproductive health services (SRH) during COVID-19 pandemics. Second, we searched for guideline databases using text words.

The association and organization selected were based on input from authors and consultations from 23 expertise in different areas of reproductive health and snowball technique applied to make the search as comprehensive as possible. To identify reproductive health experts, we did a limited search on MEDLINE to identify authors of papers published on reproductive health. We used email to reach identified authors for the survey. Finally, we developed a search strategy to look at any relevant emerging practice recommendations missed on the website and guideline database search or not endorsed by associations and organizations. We searched the following databases: MEDLINE, EMBASE, and Google Scholar. The reference lists of all included records were screened for additional studies. Text words and Mesh terms were used to develop the search strategy (Table 2 in Appendix 1, Search strategy). Electronic search and screening of records against inclusion criteria were done by two independent individuals between April 1 to April 30, 2020.

### Inclusion criteria

The report included in this scoping review was prepared based on the JBI manual of the scoping review framework [10]. We considered the following inclusion criteria:

### Population

This review considered adolescent girls, reproductive-age women, pregnant women, women seeking abortion services, health care providers, health managers, and health care institutions.

### Concept

The review considered records addressing service delivery approaches and recommendations on Antenatal care (ANC), labour and delivery, postnatal care (PNC), contraceptive service, safe abortion service, management of rape survivors, and Assisted reproductive technology (ART) services during the COVID-19 pandemic.

### Context

The review considered worldwide documents/records addressing antenatal care (ANC), labour and delivery, postnatal care (PNC), contraceptive service, safe abortion service, clinical management of rape survivors, and Assisted reproductive technology (ART) services during the COVID-19 pandemic.

#### Types of documents/records

We included records labeled guidelines, or recommendations, or consensus, or practice parameters, or position papers on SRH service practice during the COVID-19 pandemics. The search is limited to English and 1 year (considering the duration of the outbreak to be after December 2019).

#### Data extraction and synthesis

Data extraction was done by two independent persons using a validated tool that was developed to record the key information of the source that’s relevant to the review question. The data extraction tool was developed for guideline related documents, consensus statements, and practice recommendations. Types of the document and summary of recommendations were extracted. The difference between the two authors on data extraction was resolved by discussion. Data were extracted for the following practice areas: antenatal care, labour and delivery, postnatal care, safe abortion care, contraception service, gender-based violence, and assisted reproduction (Supplementary material, S1 and S1 documents). We looked for service delivery organization changes, new position statements, and guidelines on these services areas concerning the COVID-19 pandemic. We categorized identified guidelines or practice recommendations according to service delivery thematic areas and the findings were described narratively.

## Results

**Fig. 1:**
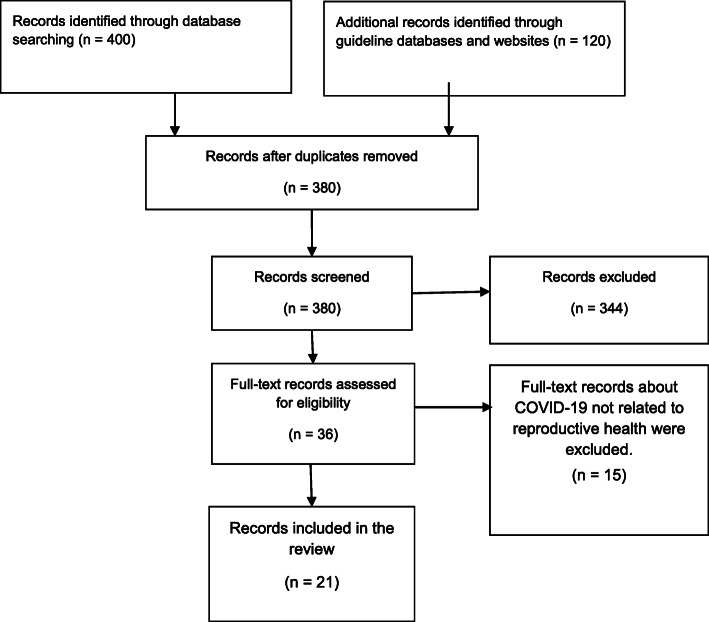
Prisma flow of record/documents selection process

We reviewed 24 websites, four guideline databases, MEDLINE, EMBASE, and Google Scholar. The search yielded a total of 520 records. After removing duplicates, 380 documents were retained for further examination. After screening the titles and abstracts, 36 papers were retained for full-text review. Based on pre-defined inclusion criteria, 21 records were included in the scoping review (Fig. 1). Six of the included records had international scope while four, six, two, two, and one where America, Europe, Australia, Canada, and Africa based guidelines and practice recommendations.

### Characteristics of included records (guidelines and practice recommendations)

Identified recommendations are categorized into service thematic areas: Antenatal Care (ANC), labour and delivery, Postnatal Care (PNC), abortion, contraception, safe abortion, gender-based violence, and assisted reproduction (Table 1).

**Table 1:** Characteristics of identified records and common practice recommendations areas

Practice area and common recommendations		Number of records	Sources and name of the record	Types of the document (guideline, commentaries, position statements)
Antenatal care	Pre-triage (screening) at visit	6	RCOG, ACOG, MFM Guidance, WHO, SMFM, NHS	Guidelines, commentaires
	Mode of service provision (telehealth, virtual, home)	7	FIGO, RCOG, ACOG, MFM Guidance, WHO, SMFM, NHS	Guidelines, position statements commentaires
	Schedule of visits	3	RCOG, SMFM, RCM	Guidance & position statements
	Attendant with pregnant mother (limit/screen status)	3	RCOG, FIGO, MFM guidance	Guidance
Labor and delivery	Place of birth (community/home/mid-wife led freestanding units)	4	RCOG, ICM, NHS, RCM	Guidance & position statements
	Labor companion (allow/limit at labor wards)	2	NHS, MFM Guidance	Guidance & Position statements
Post-natal care	Early Discharge	1	ACOG	Commentary
	Post-natal visits (virtual/ home based)	4	ACOG, RCOG, ICM, WHO	Guidance, Commentary
Obstetric Referral pathways (modifications/organization)		3	RCOG, RCM, WHO	Guidance
Safe abortion	No-touch /no-test early medication abortion.	11	FIGO, RCM, SOGC, RCOG, RANZCOG, NAF, IPPF, UNFPA, MSI, FSRH, BSACP	Guidance, position statements, and commentaries.
	Minimum contact second-trimester medication abortion.	11	FIGO, RCM, SOGC, RCOG, RANZCOG, NAF, IPPF, UNFPA, MSI, FSRH, BSACP	Guidance, position statements, and commentaries
	Minimum contact surgical abortion.	11	FIGO, RCM, SOGC, RCOG, RANZCOG, NAF, IPPF, UNFPA, MSI, FSRH, BSACP	Guidance, position statements, and commentaries
Contraception	Self-serving contraception methods.	9	FIGO, WHO, RCM, RCOG, FSRH, IPPF, UNFPA, MSI, RANZCOG	Guidance, position statements, and commentaries
	Extended use of long-term contraceptives	7	RCM, RCOG, FSRH, IPPF, UNFPA, MSI, RANZCOG	Guidance, position statements, and commentaries
	Minimum contact long term contraceptives.	8	FIGO, RCM, RCOG, FSRH, IPPF, UNFPA, MSI, RANZCOG	Guidance, position statements, and commentaries
Gender-based violence	Medical, legal, and policy mechanisms for victims should be in place.	8	UNFPA, WHO, FIGO, RANZOG, RCOG, RCM, FSRH and UN Women	Position statements
Assisted reproduction	No new cycle during pandemics except fertility preservation and freeze all embryos.	5	ESHRE, BFS, FSA, HFEA, SASREG and ASRM	Position statements and commentaries.

### Antenatal care

Antenatal care services are one of the essential services that the WHO recommends being given during pandemics [WHO]. One of the major focus areas of the guidelines and consensus statements is on antenatal care provision during the COVID-19 pandemic.

#### Pre-triage for ANC

In circumstances where a pregnant mother presents for a face to face care most of the societies including FIGO recommend screening (triage) at the entrance into the health facility [4, 11–15]. The MFM guidance advises on even an earlier pre-triage with phone communication while the patient is at home before she visits ANC clinics [MFM guidance]. Some even recommend screening of attendants too.

#### Mode of ANC provision

The mode of ANC services delivery should be modified, and innovative ways of care provision are recommended with due consideration of individualized care plan (in eight of the reviewed 12 documents) [4, 12–18]. In low-risk mothers, telehealth (voice or video calls) is a viable option for delivering prenatal care as well as triaging women before they present to the clinic. Remote access options such as home antenatal service provision by community health care workers are also suggested in WHO, RCOG, and SMFM guidelines [4, 11, 14]. But face to face care provision is advised in high-risk pregnancies and women with emergency conditions where physical examination and other clinical/laboratory tests might be needed [12, 16].

#### ANC schedules

There is no clear recommendation in any of the guidelines and consensus statements regarding modifications in the timing of 1st visit, subsequent visits, and the total number of ANC visits. But omitting or virtual visits are recommended by the RCOG and RCM [11, 19], while standard schedules are advised in high-risk mothers by the SMFM [14]. The RCOG guidance on antenatal care services and ultrasound in the COVID-19 pandemic suggests that local practice should determine re-booking [11]. Repeat visits might be scheduled using telehealth. In three of the guidelines, it is suggested that pregnant mothers present for prenatal visits alone or just with a single screen-negative attendant [11, 12, 15].

### The extent of prenatal care services

There are no recommendations on modifications to the extent of service provision in the standard prenatal care. The only guideline addressing the issue is the RCOG labor, delivery, and postnatal guidance in the COVID-19 pandemic which states scans and antenatal appointments and other investigations should be provided within a single visit one-stop clinic [11].

### Obstetric referral pathways

One of the health service challenges encountered during any pandemics is how to effectively sustain functional referral pathways, especially in low resource settings. The joint RCOG/RCM Guidance for provision of midwife-led settings and home birth in the evolving coronavirus (COVID-19) pandemic states there is good evidence to inform transport for complications and obstetric emergencies. Solutions are likely to be context-specific, dependent on e.g. urban/rural context, and the extent of pressure on the ambulance services [19]. The RCOG guidance on labor/delivery and postnatal care states obstetric antenatal referrals can be triaged locally by a consultant with a telephone appointment to discuss a proposed plan of care with the woman [16]. It is advised by the WHO that instituting targeted referral and counter-referral criteria and processes are crucial to keeping the system from becoming overwhelmed by labour and delivery [4].

### Labor and delivery

#### Induction and elective cesarean delivery (CD)

There is limited guidance on the induction of labor in the face of the COVID-19 pandemic. The RCOG recommends labor induction in low-risk mothers can be considered as an outpatient department (OPD) to ease the burden on inpatient services [16]. The NHS advises that elective procedures (including) be done as planned to avoid burden on emergency services [17].

#### Place of delivery

In institutional deliveries limiting the number of attendants is recommended but with due consideration of making sure that there is always a family member around in emergencies. A single preferably screen negative labor companion is recommended in 2 of the guidelines [12, 17].

The COVID-19 pandemic will strain labor and delivery services if tertiary centers are overwhelmed with the care of non-pregnant patients with COVID-19 infection. Some maternal health service providers might also be called to provide care in non-obstetric settings. There is also a concern for a healthy woman giving birth in a facility acquiring the COVID-19 infection. Hence, some of the guidelines have addressed the issue of the place of birth in the face of the COVID-19 pandemic. The International Confederation of Midwives (ICM) and the Royal College of Midwives (RCM) support community birth (home birth) for healthy women and newborn infants if there are appropriate mid-wife staffing and referrals are facilitated in obstetric emergencies [19–21]. Where these are not available, it may be necessary to modify available services, seeking at all times to maximize the provision of a safe and positive birth experience to all women [21]. The NHS Clinical guide for the temporary reorganization of intrapartum maternity care during the coronavirus pandemic has put 4 options of childbirth: homebirth, alongside midwifery-led unit, freestanding midwifery-led unit, and obstetric unit. Freestanding mid-wife led delivery services are forwarded as viable options of childbirth by both NHS and RCOG guidance [17, 21]. But this usually requires a response from an ambulance service, which may also currently be stretched. This means transfers from home to the hospital may not be sufficiently quick to ensure the safety of mother and baby [17].

### Post-natal care

Modification of postnatal services is recommended with fewer visits and provision of care with telehealth. Telehealthcare even can extend to those patients who have undergone surgeries. Generally, earlier discharge of mothers with uncomplicated deliveries is recommended (immediate or less than 24 h in those after vaginal delivery and after 24 h in cesarean section [13].

In the presence of community-based health worker’s homes, postnatal care provision is another option suggested by WHO [4]. The RCOG postnatal care guidance recommends for most women telephone or home visits may be preferable to community clinic visits to comply with social distancing. Face to face visiting is recommended for women with Known psycho-social vulnerabilities, operative birth, premature/low birth weight baby, and other medical or neonatal complications [11]. But ACOG advisory commentary suggests that phone call consultations and video conferencing with inspection of photos of wound site can be done in women who have undergone surgery [13].

### Contraception service

#### For women already on contraception

Telemedicine and self-care family planning methods were recommended consistently. Self-care family planning methods include contraceptive pills, self-injectables, subcutaneous depo shots, condoms, vaginal rings, and fertility awareness methods [WHO, FIGO, RCOG, RCM, SOGC, RANZOG, IPPF, UNFPA, MSI, and FSRH] [3, 22–26].

There are consistent position statements that recommend combined hormonal contraception (CHC) and progesterone-only pills (POP) users to continue 6–12 months without visits and rechecking body mass index (BMI) and blood pressure. Depot medroxyprogesterone acetate (DMPA) users can switch to available progesterone-only pills (POP) to avoid face to face contact [3, 22, 27, 28]. For long term contraceptive user’s options of extended use to avoid face to face contact is recommended. Limited evidence shows that the duration of the long-acting contraceptive effect is 2 years beyond the Food and Drug Administration (FDA)-approved duration [29]. Depending on that evidence many associations and organizations practice recommendations [FIGO, RCOG, RCM, SOGC, RANZOG, IPPF, UNFPA, MSI, and FSRH] advised delaying removal of implants and IUCD during the pandemic crisis unless a series of side effect happens or wants to get pregnant [3, 22, 25, 28, 30, 31].

#### New contraception starters

Telemedicine and self-care family planning with remote assessment and prescription of CHC, POP for 6–12 months, and self-injectable contraception were consistently recommended. However, administration of DMPA or insertion of implants or intrauterine devices to be considered where concerns about adherence, individual intolerance of oral contraceptives, or use of teratogens make longer-acting reversible contraception the only suitable option. Pre-procedure assessment and information-giving remotely to minimize face-to-face contact time (minimum contact service) with healthcare professionals were recommended [WHO, FIGO, RCOG, RCM, SOGC, RANZOG, IPPF, UNFPA, MSI, and FSRH]. Optimal use of contact points, such as expanding post-partum family planning with a special focus on long-acting reversible contraception was recommended [FIGO, RCOG, RCM, FSRH, MSI, and UNFPA].

#### Emergency contraception (EC)

Remote assessment of requirements and choice of EC. Oral emergency contraception remote prescription or provision without prescription or Cu-IUD provision with minimum face to face contact is recommended [RCOG, RCM, FSRH, BSACP, FIGO].

### Safe abortion service

All records (practice recommendations and position papers or commentaries) consistently recommend screening for COVID-19 symptoms from remote before face to face contact or during remote early medication abortion without face to face contact. There were several recommendations on no-touch/no-test early medication abortion protocol [2, 3, 27, 30, 32]. The no-touch protocol depicts pathways to minimize COVID-19 exposure to patients and staff by organizing early medical abortion services to be delivered via video or teleconferencing /telemedicine and delivery of a treatment package [2, 27, 30]. The treatment package includes mifepristone, misoprostol, ibuprofen, and self-care family planning. The no-touch/no-test protocol is self-medication abortion in early pregnancy without pre-procedure ultrasound and blood testing. The guideline also indicated that for women in self-isolation because of exposure to COVID-19 no-touch early medication abortion can be arranged similarly at home. If face to face contact care is a must for COVID-19 exposed women, the guideline recommends that it should be booked when the isolation period is over unless the gestation is uncertain, and the delay may result in a woman not being able to access abortion in which face to face contact must be arranged with full personal protective measures [27]. There is no specific protocol recommended for second-trimester medication abortion (above 12 weeks), but professional association and organizations position papers consistently recommend the utilization of telemedicine for digital patient education and counseling to reduce waiting for periods and extent of face to face contact (minimal contact service) [4, 30, 32, 33].

For surgical abortion position papers and practice, recommendations focus on minimum contact procedures by remote digital patient education, counseling, and evaluation. The other focus practice recommendation is increasing safety during the procedure by limiting the number of people in the procedure room, appropriate use of personal protective equipment’s and decontaminate area after the procedure as per the recommendation [30, 32, 33]. The practice recommendations also include surgical facemask and sanitizer or hand washing for women. Vacuum aspiration, dilatation, and evacuation or dilatation and curettage are not aerosol-generating procedures unless done by general anesthesia [34]. Therefore, these procedures don’t require full personal protective equipment like N95, but abortion provides should screen all patients before the procedure and use standard precautions. Where possible and feasible it’s also highlighted to perform the procedures under local anesthesia or intravenous sedation or spinal anesthesia to avoid the need for general anesthesia [27, 30, 32, 34]. It recommended consistently that follow-up visits are not required in all conditions and were needed to be done remotely by telemedicine.

### Gender-based violence (GBV)

It is recommended that medical, legal, and policy mechanisms for victims of gender-based violence remain in place during the pandemic crisis. Access to clinical care (medical evaluation and management) for rape survivors is recommended to be maintained 24/7 with necessary modifications in referral pathways to increase access [UNFPA, WHO, FIGO, RANZOG, RCOG, RCM, FSRH, and UN Women] [3, 4, 33, 35, 36].

### Assisted reproductive technology (ART)

It recommended that assisted reproduction (including diagnostic procedures for infertility) shouldn’t be started during the pandemics except in cases of urgent fertility preservation such as in oncology patients, the cryopreservation of gametes, embryos, or tissue can still be considered [ESHRE, BFS, FSA, HFEA, SASREG, and ASRM]. For those already on treatment it’s recommended to freeze all for later embryo transfer [37–42].

## Discussion

In this review, we attempted to locate documents in the form of guidelines, consensus statements, best practice statements, and standards of practice indicating directions on how reproductive health service during COVID-19 pandemics. We searched guideline databases, MEDLINE, EMBASE, and Google Scholar and the website of international professional associations and organizations working on sexual and reproductive health. Several international associations and organizations have declared service related to reproductive health, including contraception and safe abortion care as essential health service to continue during the COVID-19 pandemics [WHO, ACOG, RCOG, FIGO, RCM, SOGC, RANZCOG, SFP, NAF, IPPF, UNFPA, MSI, BSACP, and FSRH].

There were consistent consensus statements and recommendations that there should be access to routine SRH services like ANC, PNC, essential newborn care, breastfeeding support, contraception service, safe abortion care, and clinical management of rape survivors during the COVID-19 pandemics. This is very important to maintain service delivery and prevent indirect mortalities and morbidities from the pandemic as in the past Ebola virus outbreak in 2013–2016 in Western Africa. For example, according to an analysis of data from Sierra Leone’s Health Management Information System, decreases in maternal and newborn care due to various reasons related to the Ebola outbreak contributed to an estimated 3600 maternal deaths, neonatal deaths, and stillbirth, a quantity that approaches the number of deaths directly caused by the Ebola virus in the country [9]. Evidence also shows that the number of antenatal care visits and facility deliveries after the Ebola epidemic in Guinea had not recovered to prior levels after 6 months, suggesting the possibility of epidemic sustained effects on the different reproductive health care [43]. Therefore, the lessons from past outbreaks like Ebola exemplify the necessity of implementing evidence-based guidelines and recommendations to maintain the continuation of reproductive services during the COVID-19 pandemic. The practice recommendations identified by the current review focus on minimizing patient and staff exposure to COVID-19 by utilizing telemedicine or digital health and includes the following: Pre-triage (screening) of all clients visiting a health facility for COVID-19, modification of antenatal care provision from face-to-face to telehealth (voice or video calls), if possible, a one-stop clinic service provision with clinical examination and lab tests were done on the same visit, instituting targeted obstetric referral and counter-referral criteria, re-organization of intrapartum care and modification of postnatal services with earlier discharge, community-based (mid-wife led) or home births with functional back-up referral systems and modification (reorganization) of obstetrical referral pathways, no-touch/no-taste early medication abortion or minimum contact safe abortion care and self-serving family planning.

The utilization of digital health is well recognized in increasing access to service. The WHO recommends self-management of different conditions including abortion less than 12 weeks provided that women had easy access to information [44]. However, the utilization of telemedicine might be difficult in many low- and middle-income countries in which awareness and utilization of digital health are very low. This might exacerbate the already existing health inequity. To overcome such gaps awareness creation campaigns and utilization of community health workers are very imperative.

For women already on combined hormonal contraception (CHC) and progesterone-only pills (POP), it’s recommended to continue 6–12 months without rechecking body mass index (BMI) and blood pressure during the pandemics to reduce exposure to COVID-19. Furthermore, it is recommended to use options of extended use of long-term contraceptive methods to avoid face to face contact during the pandemics. Additionally, during COVID-19 pandemics every stakeholder should make sure medical, legal, and policy mechanisms for victims of gender-based violence are in place. However, many recommend not to start a new assisted reproduction cycle during the pandemic except for fertility preservation and to freeze all embryos for those who were already on treatment.

### Strengths and limitations

The review located and summarized contemporary international and national guidelines and recommendations on reproductive health service organization and service delivery during COVID-19 pandemics. This is the very timely and important review that guides service delivery amid COVID-19. The review will also be used as a bench mark to frame the reproductive service delivery approach in cases of any future pandemic or epidemic. We searched professional associations and organization’s websites, guideline databases, and databases to make the search as comprehensive as possible. The selection of professional associations and organization’s websites was based on the author’s consensus and recommendation by reproductive health experts which might have introduced selection bias. Our review included only documents published in English which might result in missing relevant non-English publications. Additionally, most of the documents included didn’t pass through a rigorous guideline development process because of the nature of the pandemic. Furthermore, we did not perform a formal critical appraisal of individual records for this systematic scoping review. The review included records published until April 30/2020 which might result in missing records that are published after that. There is geographical variation in the records included in this review which might be because of the timing of the spread of COVID-19 to different continents. Hence, we plan to update the review within 6 months of publication to include missed records and generate more comprehensive evidence.

## Conclusions

### Implications for practice

There were consistent consensus statements and recommendations that there should be access to sexual and reproductive health services during the COVID-19 pandemics with the concerted effort of service re-organization. The practice recommendations focus on innovative ways of service provision to minimize patient and staff exposure to COVID-19 as well as alleviate the burden on the health care system. These include utilizing telemedicine and community/home-based care or self-care.

### Implications for research

Most of the documents that are included in this review didn’t pass through a rigorous guideline development process because of the nature of the pandemic. New evidence is evolving with time as the duration of the pandemic extends. Hence, we recommend primary studies and systematic reviews to generate evidence on the impact of new practices, map and document best practice implementations.

## Supplementary Information

**Additional file 1.** Data extraction tool ANC, labour and delivery, and postnatal care.

**Additional file 2.** Data extraction tool safe abortion, contraception, GBV, and ART service.

## Data Availability

All the data set used were included in the manuscript and supplementary materials.

## Appendix 1

### Search Strategy

We searched website of the following associations and organizations: World Health Organization (WHO), America College of Obstetrics and Gynecology (ACOG), Royal College of Obstetrics and Gynecology (RCOG), International Confederation of Midwives (ICM), Royal College of Midwives (RCM),International Society of Ultrasound in Obstetrics and Gynecology (ISUOG),International Federation of Obstetrics and Gynecology (FIGO),Society of Maternal and Fetal Medicine (SMFM),Society of Obstetrics and Gynecology of Canada (SOGC),RANZCOG (The Royal Australian and New Zealand College of Obstetricians and Gynecologists), National Health Service (NHS), UNICEF (United Nations International Children’s Emergency Fund), Faculty of Sexual and Reproductive Healthcare (FSRH), British Society of Abortion Care Providers (BSACP), National Abortion Federation (NAF), Society of Family Planning (SFP),United Nations Population Fund (UNFPA), International Planned Parenthood Federation (IPPF), Marie stopes International (MSI), European Society of Human Reproduction and Embryology (ESHRE),American Society for Reproductive Medicine (ASRM),Human Fertilization and Embryology Authority (HFEA),British Fertility Society (BFS), Chinese Obstetricians and Gynecologists Association (COGA), The fertility Society of Australia (FSA), African Federation of Obstetricians and Gynaecologist (AFOG), Southern African Society of Reproductive Medicine and Gynecological Endoscopy (SASREG) and the Asia & Oceania Federation of Obstetrics & Gynecology (AOFOG). The guideline databases searched were: Turning Research into Practice (TRIP) database, Guideline International (GIN) library, National Guideline Clearinghouse (NGC), and National Institute for Health and Clinical Excellence (NICE).

**Table 2:** Search Strategy 1. The search was conducted on April 18, 2020, MEDLINE (Ovid).

Number	Search Query	Result.
1	COVID-19 [tw] OR Sars cov-2[tw] OR Pandemics [tw] OR epidemics [tw] AND maternal care [tw] OR reproductive service [tw] OR antenatal care [tw] OR labour [tw] OR delivery [tw] OR abortion [tw] OR contraception [tw] OR family planning [tw]	92,0931
2	Limit 1 to English AND Human AND 1 year.	400

## Abbreviations

SRH: Sexual and Reproductive Health
ANC: Antenatal Care
PNC: Postnatal care
WHO: World Health Organization
ACOG: America College of Obstetrics and Gynecology
RCOG: Royal College of Obstetrics and Gynecology
ICM: International Confederation of Midwives
RCM: Royal College of Midwives
ISUOG: International Society of Ultrasound in Obstetrics and Gynecology
FIGO: International Federation of Obstetrics and Gynecology
SMFM: Society of Maternal and Fetal Medicine
SOGC: Society of Obstetrics and Gynecology of Canada
RANZCOG: The Royal Australian and New Zealand College of Obstetricians and Gynecologists
NHS: National Health Service
UNICEF: United Nations International Children's Emergency Fund
FSRH: Faculty of Sexual and Reproductive Healthcare
BSACP: British Society of Abortion Care Providers
NAF: National Abortion Federation
SFP: Society of Family Planning
UNFPA: United Nations Population Fund
IPPF: International Planned Parenthood Federation
MSI: Marie stopes International (MSI)
ESHRE: European Society of Human Reproduction and Embryology
ASRM: American Society for Reproductive Medicine
HFEA: Human Fertilization and Embryology Authority
BFS: British Fertility Society
COGA: Chinese Obstetricians and Gynecologists Association
FSA: The fertility Society of Australia (FSA)
AFOG: African Federation of Obstetricians and Gynecologist
SASREG: Southern African Society of Reproductive Medicine and Gynecological Endoscopy
AOFOG: Asia & Oceania Federation of Obstetrics & Gynecology
TRIP: Turning Research into Practice
GIN: Guideline International
NGC: National Guideline Clearinghouse
NICE: National Institute for Health and Clinical Excellence
MSI: Marie Stopes International
