# Supplementary material for: Guidelines and best practice recommendations on reproductive health services provision amid COVID-19 pandemic: scoping review
Source: BMC Public Health. 2021 Feb 3;21:276. doi: 10.1186/s12889-021-10346-2 (PMC7856605; doi:10.1186/s12889-021-10346-2)
Supplement: Supplementary file 1 — Additional file 1. Data extraction tool ANC, labour and delivery, and postnatal care. [file 12889_2021_10346_MOESM1_ESM.docx]

Data extraction format: ANC,PNC and labour and delivery: Scoping review

|  | 1 | 2 | 3 | 4 | 5 | 6 | 7 | 8 | 9 | 10 | 11 | 12 |
| --- | --- | --- | --- | --- | --- | --- | --- | --- | --- | --- | --- | --- |
| ***Antenatal care, labor & delivery*** | | | | | | | | | | | | |
|  | [RCOG US Antenatal](file:///C:\Users\user\Desktop\covid-19%20RH%20SERVECES%20REVIEW\2020-03-25-covid19-antenatal-screening.pdf) | [RCOG L/D PNC guideline](file:///C:\Users\user\Desktop\covid-19%20review\2020-03-30-guidance-for-antenatal-and-postnatal-services-in-the-evolving-coronavirus-covid-19-pandemic-200409.pdf) | [ICM](file:///C:\Users\user\Desktop\covid-19%20RH%20SERVECES%20REVIEW\icm-statement_upholding-womens-rights-during-covid19-5e83ae2ebfe59.pdf) | [RCOG mid wife led](file:///C:\Users\user\Desktop\covid-19%20RH%20SERVECES%20REVIEW\RCOG%20guidance-for-provision-of-midwife-led-settings-and-home-birth-in-the-evolving-coronavirus-covid-19-pandemic%20(1).pdf) | [RCM optimizing maternity services](file:///C:\Users\user\Desktop\covid-19%20RH%20SERVECES%20REVIEW\RCOG%20guidance-for-provision-of-midwife-led-settings-and-home-birth-in-the-evolving-coronavirus-covid-19-pandemic%20(1).pdf) | [ACOG commentary](file:///C:\Users\tolul\Downloads\covid-19%20review\ACOG%20RESPONDING%20TO%20CORONA.pdf) | [MFM](C:\\Users\\user\\Desktop\\covid-19 RH SERVECES REVIEW\\MFM GUIDANCE FOR COVID, AJOG.pdf)  [Guidance](C:\\Users\\user\\Desktop\\covid-19 RH SERVECES REVIEW\\MFM GUIDANCE FOR COVID, AJOG.pdf) | [WHO](file:///C:\Users\user\Desktop\covid-19%20review\WHO-2019-nCoV-essential_health_services-2020.1-eng.pdf) | [SMFM](file:///C:\Users\user\Desktop\covid-19%20review\SMFM%20COVID19-_updated_3-17-20_PDF.pdf) | [NHS](C:\\Users\\user\\Desktop\\covid-19 RH SERVECES REVIEW\\NHS reorganization of intrapartum-maternity-care-9-april-2020.pdf)  [SERVICE REORG](C:\\Users\\user\\Desktop\\covid-19 RH SERVECES REVIEW\\NHS reorganization of intrapartum-maternity-care-9-april-2020.pdf) | [FIGO](file:///C:\Users\user\Desktop\covid-19%20review\FIGO%20SPECIAL%20ARTICLE.docx) | [RCOG covid](C:\\Users\\user\\Desktop\\covid-19 review\\2020-04-09-coronavirus-covid-19-infection-in-pregnancy.pdf)  [inf preg](C:\\Users\\user\\Desktop\\covid-19 review\\2020-04-09-coronavirus-covid-19-infection-in-pregnancy.pdf) |
| Screening for corona (pretriage) | recommended | NA | NA | NA | NA | Recommended including attendants | Phone triage as well onsite | Triage of all clients | Triage at visit |  | At entrance to the clinic |  |
| Mode of ANC provision visits  (Telehealth, virtual, phone, community based or usual way?) | Phone based | Telephone or remote assistance by care provider  Low risk virtual | NA | NA | NA | Adjustment  Based on clients condition | Most should be telehealth / remotely  unless urgent | Out reach mechanisms considered | Telehealth Or Other remote access links | Telephone | replace with virtual visits either with video phone calls | Units should rapidly seek to adopt teleconferencing and videoconferencing capability and consider what appointments can be conducted remotely |
| Subsequent visits | Local service decide on re booking | Virtual visit or omit | NA | NA | Virtual technology |  | NA | NA | Standard care for high risk | NA | NA | NA |
| ANC attendant with pregnant mother | Preferably alone or single attendant | Na | NA | NA | NA | NA | screen Neg. attendant | NA | NA | NA | Earlier call to inform or alone |  |
| ***Labor and Delivery*** | | | | | | | | | | | | |
| Delivery by TBAs (community or home birth) | NA | NA | support community birth for healthy women | Midwife Led Delivery Services advised | Community Based Care Services | NA | NA | NA | NA | freestanding midwifery units and home birth teams | NA | NA |
| Birth companion | NA | NA | NA | NA | NA | NA | Single screened , no change of attendants | NA | NA | Asymptomatic partner | NA | NA |
| ***Postnatal care*** | | | | | | | | | | | | |
| Postpartum discharge | NA | NA | NA | NA | NA | Early (<24hrs) in SVD and  24-48 after CD | NA | NA | NA | NA | NA | NA |
| Modified ways of service provision (tele-health?) |  | - face to face visiting for women with:  Known psycho-social vulnerabilities,  Operative birth,  Premature/low birthweight baby  Other medical or neonatal complexities  - Telephone or  Home visits may be preferable to community clinic visits to comply with social distancing | OTHER SERVICES SHOULD CONTINUE | NA | NA | All telehealth including Cd wound failures (Photos) | NA | Home PNC | NA | NA | NA | NA |
